# β-Defensin-2 Protein Is a Serum Biomarker for Disease Activity in Psoriasis and Reaches Biologically Relevant Concentrations in Lesional Skin

**DOI:** 10.1371/journal.pone.0004725

**Published:** 2009-03-06

**Authors:** Patrick A. M. Jansen, Diana Rodijk-Olthuis, Edward J. Hollox, Marijke Kamsteeg, Geuranne S. Tjabringa, Gys J. de Jongh, Ivonne M. J. J. van Vlijmen-Willems, Judith G. M. Bergboer, Michelle M. van Rossum, Elke M. G. J. de Jong, Martin den Heijer, Andrea W. M. Evers, Mieke Bergers, John A. L. Armour, Patrick L. J. M. Zeeuwen, Joost Schalkwijk

**Affiliations:** 1 Department of Dermatology and Nijmegen Centre for Molecular Life Sciences, Radboud University Nijmegen Medical Centre, Nijmegen, The Netherlands; 2 Department of Genetics, University of Leicester, Leicester, United Kingdom; 3 Department of Endocrinology and Department of Epidemiology and Biostatistics, Radboud University Nijmegen Medical Centre, Nijmegen, The Netherlands; 4 Department of Medical Psychology, Radboud University Nijmegen Medical Centre, Nijmegen, The Netherlands; 5 Institute of Genetics, University of Nottingham, Nottingham, United Kingdom; The University of Queensland, Australia

## Abstract

**Background:**

Previous studies have extensively documented antimicrobial and chemotactic activities of beta-defensins. Human beta-defensin-2 (hBD-2) is strongly expressed in lesional psoriatic epidermis, and recently we have shown that high beta-defensin genomic copy number is associated with psoriasis susceptibility. It is not known, however, if biologically and pathophysiologically relevant concentrations of hBD-2 protein are present *in vivo,* which could support an antimicrobial and proinflammatory role of beta-defensins in lesional psoriatic epidermis.

**Methodology/Principal Findings:**

We found that systemic levels of hBD-2 showed a weak but significant correlation with beta defensin copy number in healthy controls but not in psoriasis patients with active disease. In psoriasis patients but not in atopic dermatitis patients, we found high systemic hBD-2 levels that strongly correlated with disease activity as assessed by the PASI score. Our findings suggest that systemic levels in psoriasis are largely determined by secretion from involved skin and not by genomic copy number. Modelling of the *in vivo* epidermal hBD-2 concentration based on the secretion rate in a reconstructed skin model for psoriatic epidermis provides evidence that epidermal hBD-2 levels *in vivo* are probably well above the concentrations required for *in vitro* antimicrobial and chemokine-like effects.

**Conclusions/Significance:**

Serum hBD-2 appears to be a useful surrogate marker for disease activity in psoriasis. The discrepancy between hBD-2 levels in psoriasis and atopic dermatitis could explain the well known differences in infection rate between these two diseases.

## Introduction

Psoriasis is a highly prevalent inflammatory skin disease that has both environmental and genetic components to its etiology [1], [2]. Genetic evidence for an (auto)immune basis of psoriasis is provided by the well-known association of the disease with the HLA-Cw6 gene [3] and the recently discovered associations with IL12B and IL23R [4], [5]. Lesional psoriatic skin is characterized by various morphological abnormalities of the epidermis, and a cellular infiltrate of activated T-cells. There are several arguments to invoke an important role of activated T-cells such as the oligoclonal T-cell expansion in psoriatic skin [6] and the therapeutic efficacy of T-cell directed drugs such as cyclosporin A and some of the biologics that are currently available. Recent evidence also points to a role of other cell types such as plasmacytoid dendritic cells [7], and cytokine networks associated with cells from the adaptive and innate immune system [1], [8]. Based on clinical studies in humans and experimental studies in mice, several of these cytokines have been identified including IL-1, TNFα, interferon-γ and IL-6. In the epidermis a regenerative epidermal differentiation program is induced that includes hyperproliferation and expression of genes such as cytokeratin 16 (CK16), SKALP/elafin, psoriasin and hBD-2 [9], [10]. Expression of these genes is to some extent specific for psoriasis, as they are expressed at low levels, if at all, in lesional atopic dermatitis skin [11]–[13].

Recent findings from various labs including our own have indicated that polymorphisms of genes that are expressed in the epithelium, but not necessarily in immunocytes, could also be risk factors for inflammatory skin diseases such as atopic dermatitis and psoriasis [14]–[17]. This finding was further supported at the cellular level when we found cell-autonomous differences between keratinocytes from psoriasis and atopic dermatitis patients [18]. From these studies we concluded that psoriatic keratinocytes are programmed to secrete large amounts of host defense proteins such as beta-defensins, in response to Th1 or Th17 cytokines.

Beta-defensins are secreted peptides of low molecular weight ranging from 3 to 5 kDa. These peptides, which are expressed by epithelia, possess a broad spectrum of antimicrobial activity against both gram-positive and gram-negative bacteria, fungi and viruses [19]. Besides antimicrobial activity, they also exhibit pro-inflammatory properties as chemoattractants for memory T-cells, immature dendritic cells, mast cells and neutrophils [20]–[22]. These peptides, encoded by the *DEFB* genes, are present in three main gene clusters, two on chromosome 20 and one on 8p23.1. The cluster on 8p23.1 contains eight beta-defensin genes of which seven (all but *DEFB1*) are on a large repeat that is variable in copy number. In humans up to 12 copies of this repeat have been found, with a mode of four copies per diploid genome [23]. Of the eight beta-defensin genes located on 8p23.1, human beta-defensin-1 (hBD-1) protein, (encoded by *DEFB1*) and hBD-3 (encoded by *DEFB103*) are constitutively expressed at low levels in skin [24]. hBD-2 (encoded by *DEFB4*) is not expressed in normal skin but is highly expressed in psoriatic skin. hBD-4 (encoded by *DEFB104*) is less well characterized at the protein level but is found in skin by RT-PCR. hBD-2, hBD-3 and hBD-4 can be induced by cytokines and bacterial lipopolysaccharides in various epithelial cell types [25]. It has also been shown that antimicrobial peptides in general, and above all hBD-2, are induced in lesional epidermal cells of patients with psoriasis, compared with lesional epidermal cells of patients with atopic dermatitis and normal skin [10], [11]. These findings have been interpreted to explain the observed high infection rate in atopic dermatitis and the relatively low prevalence of bacterial and viral infections among psoriasis patients [26].

In addition to their direct antimicrobial activity, the chemotactic properties of antimicrobial proteins like LL-37 and beta-defensins are thought to amplify leukocyte recruitment [27], thereby contributing to an effective antimicrobial response. In non-infectious inflammatory diseases like psoriasis, beta-defensins could augment the influx of T-cells and dendritic cells, and thereby contribute to chronicity and sustained disease. Although the antimicrobial and chemotactic activities *in vitro* have been well documented, there are no quantitative data on *in vivo* concentrations of beta-defensins to substantiate a role of these molecules in host defense or inflammation. In this study we have made a detailed analysis of systemic and epidermal defensin concentrations. We show that both genetic factors and disease activity determine defensin protein expression. Evidence is provided that the *in vivo* concentration of hBD-2 is well above the minimal concentrations that are required for biological activity *in vitro*.

## Materials and Methods

### Patients and healthy subjects

All psoriasis patients had plaque-type psoriasis diagnosed by a dermatologist. Atopic dermatitis was diagnosed by a dermatologist, according to the Hanifin criteria, and were of the extrinsic type [28]. Rheumatoid arthritis patients were diagnosed by a rheumatologist, according to the ACR criteria. Patients were recruited via the in-patient or out-patient departments of the Radboud University Nijmegen Medical Centre. Control sera and DNA samples were from the Nijmegen Biomedical Study (NBS) [29]. All controls and patients were of native European Dutch origin. Demographic data were as follows (age: mean and SD, and % female); Controls: 50±16, 59% female; Psoriasis patients: 47±13 year, 42% female; Rheumatoid arthritis patients: 59±8 years, 66% female. Available information of medication used during sample collection: 59% of the psoriasis patients were on biologicals (all TNF-blockers) and 36% were on other types of systemic medication (retinoids, MTX). Nearly all rheumatoid arthritis patients used NSAIDs, 45% was on MTX and 25% was on biologicals (various kinds, mostly TNF blockers). Blood was stored at −80°C and genomic DNA was isolated by standard procedures. Permission for these studies was obtained from the local medical ethics committee (Commissie Mensgebonden Onderzoek Arnhem-Nijmegen), and volunteers gave written informed consent. The study was conducted according to the Declaration of Helsinki principles.

### Clinical scores

As a measure of disease severity the clinical score was determined by medical specialists. For psoriatic patients the PASI (0 to 72 scale) [30], for atopic dermatitis patients the SCORAD (0 to 103 scale) [31] and for rheumatic arthritic patients the DAS28 (0 to 10 scale) [32] was used to measure disease severity. Patients were classified as being in remission, or having low disease activity, moderate disease activity or high disease activity (see table 1).

**Table 1:** Disease severity and serum hBD-2 of patients and controls

Diagnosis	Disease severity	Disease score	N	Serum hBD-2 (ng/ml)
Controls	n.a.	n.a.	70	0.21±0.17
Psoriasis	remission, PASI 0–1	0.4±0.4	3	0.64±0.36
	low, PASI 1–10	6.0±2.7	18	4.5±4.6
	moderate, PASI 10–20	15.1±2.8	12	11.5±9.1
	high, PASI>20	30.2±3.6	5	84.2±80.0
Atopic dermatitis	remission, SCORAD 0–5	0±0	2	0.10±0.01
	low, SCORAD 5–15	12.0±2.8	2	0.12±0.03
	moderate, SCORAD 15–40	27.9±9.4	6	1.05±0.95
	high, SCORAD>40	47.6±5.0	2	1.39±0.74
Rheumatoid arthritis	remission, DAS28 0–2.6	2.1±0.5	10	0.33±0.16
	low DAS28, 2.6–3.2	2.9±0.1	5	0.34±0.19
	moderate, DAS28 3.2–5.1	4.2±0.6	16	0.51±0.41
	high, DAS28>5.1	6.0±0.4	9	0.83±0.59

Serum hBD-2 levels were determined by ELISA. Analysis of variance on log transformed data showed that there was a significant correlation between disease severity and serum hBD-2 levels in psoriasis patients (p<2×10^−5^). Disease scores and serum hBD-2 concentration are given as mean and SD. n.a. not applicable.

### Immunohistochemistry

Autopsy material and skin biopsies were fixed in 4% phosphate-buffered formalin and embedded in paraffin as described before [33]. Sections of seven micrometer were stained according to the avidin biotinylated-enzyme-complex method (Vector Laboratories, Burlingame, CA) using a goat anti-hBD-2 serum (Abcam, Cambridge, UK).

### RNA extraction and real-time quantitative polymerase chain reaction

**Figure 1:**
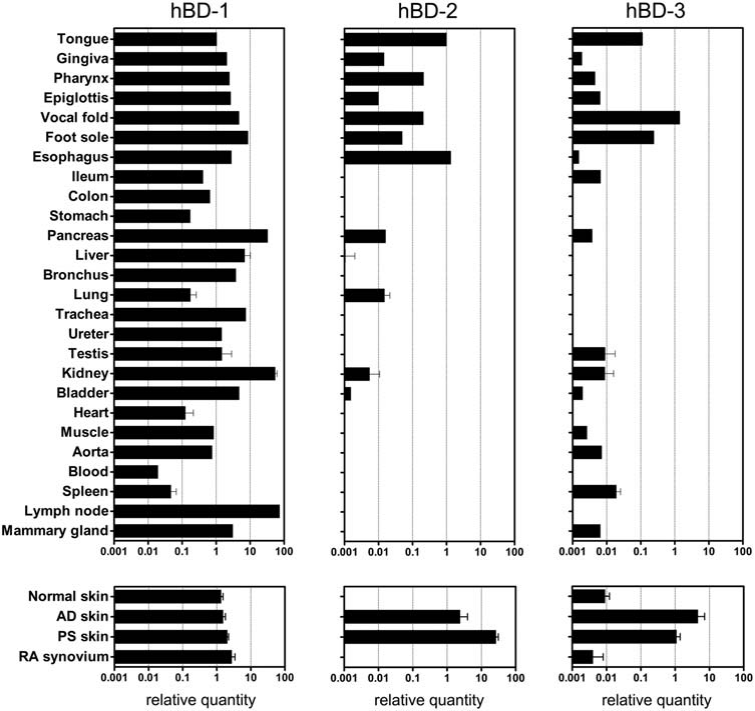
Analysis of beta-defensin mRNA expression in normal and inflamed human tissues. Quantitative real-time PCR was performed on RNA of normal human tissues (mostly obtained from one individual), purified epidermis from skin biopsies of healthy controls, psoriasis patients and atopic dermatitis patients, and inflamed synovium from rheumatoid arthritis patients. Expression of target genes was normalized to that of RPLP0. For graphical representation all values were expressed relative to hBD-2 in tongue, which was set at unity [34]. Primer sequences and efficiency of amplification are given in supplemental table S1. For details on normal human tissues see materials and methods. Bars represent mean and SD.

RNA from a variety of organs that included many epithelial tissues, was available from autopsy material of one individual. For this reason the qPCR data in figure 1 have only n = 1 for most organs. Additional RNA samples from lung (8), kidney (2), heart (1), testis (1), spleen (1) and liver (1), normal (5) and inflamed epidermis (7) and inflamed synovium (5) were obtained from different individuals and analyzed for beta defensin expression. Variability between the different samples from one type of organ was low, and similar profiles of differential expression of beta defensins 1, 2 and 3 were found. When multiple samples per organ were available, expression values are given as mean and SD in figure 1.

RNA was extracted from twenty-four different normal human tissues, punch biopsies and cultured human keratinocytes, and first strand cDNA was synthesized using an input of 1 µg of RNA with the iScript cDNA synthesis kit (Bio-Rad, Hercules, CA) according to the manufacturer's recommendation. The reverse transcriptase product was used as a template for quantitative real-time PCR amplification of genes of interest hBD-1, hBD-2 and hBD-3 and the housekeeping gene human ribosomal phosphoprotein P0 (RPLP0) with the MyiQ Single-Color Real-Time Detection System for quantification with Sybr Green and melting curve analysis (Biorad, Richmond, CA). Expression of target genes was normalized to that of RPLP0. This housekeeping gene was not found to be subject to regulation in keratinocyte cultures, irrespective of stimulation or diagnosis, and is more reliable than other reference genes such as ACTB (actin) or GAPDH (data not shown). RPLP0 levels of the various tissues did not show large differences (mean and SD of Ct values of normal human tissues was 21.7±1.6). The deltaCt values were used for statistical analysis. For graphical representation (figure 1) all values were expressed relative to hBD-2 in tongue, which was set at unity [34]. This allows comparison of all genes and all tissues for relative expression levels. Primer sequences (Biolegio, Nijmegen, The Netherlands) and efficiency of amplification are given in supplemental table S1.

### Production of recombinant hBD-2 and rabbit antiserum

A hBD-2 PCR product from cDNA derived from cultured keratinocytes was cloned into pGEX-2T vector (Amersham Pharmacia Biotech, Uppsala, Sweden), expressed as a GST-hBD-2 fusion protein in *Escherichia coli* and affinity-purified. Commercial recombinant hBD-2 (Peprotech, Rocky Hill, NJ) was cross-linked to ovalbumin, using glutaraldehyde, to increase immunogenicity. This preparation was dialysed against phosphate-buffered saline and emulsified with complete Freund's adjuvant to immunize rabbits to generate polyclonal serum. Animals were boostered with GST-hBD-2, and blood was obtained for serum preparation.

### ELISA

Affinity-purified goat anti-hBD-2 (Abcam) was used to coat 96-well microtiter plates. After blocking in 1% (v/v) bovine serum albumin, serum samples were applied in a serial 2-fold dilution range, followed by our rabbit anti-hBD-2 as a second antibody, and detection by the ABC kit (Vector). All steps were followed by appropriate washing in phosphate-buffered saline with 0.05% (v/v) Tween-20. The serum hBD-2 concentrations were read from a calibration curve of recombinant hBD-2 (Peprotech). The detection limit of this ELISA was 0.03 ng/ml, using recombinant hBD-2 (Peprotech) for calibration. Anti-hBD-2 antibodies were checked for specificity against hBD-2, and we found that recombinant hBD-1 (Abcam) and hBD-3 (Abcam) were not recognized. We found no evidence for circadian differences in serum hBD-2 in healthy individuals (data not shown). Serum hBD-2 was found to be stable upon storage at −20°C. No large differences were found for individuals from whom we had serum samples taken with a two-year interval. Commercially available ELISA kits for hBD-2 and hBD-3 were purchased from Phoenix (Belmont, CA) and performed according to manufacturer's recommendations. The detection limits for these assays were 0.05 and 0.1 ng/ml respectively, using synthetic hBD-2 and hBD-3 for calibration.

### Genotyping of defensin copy number

Genomic DNA from seventy control individuals without inflammatory skin disease was analyzed for genomic copy number. Most of these individuals were analyzed by three methods: MAPH, REDVR and PRT as described previously in great detail [16], [23], [35]. These analyses were used to obtain the best estimate of integral copy number.

### Three-dimensional reconstructed skin

Reconstructed skin was generated as described before [36]. Briefly, de-epidermized human dermis (DED) of 0.8 mm thickness and 8 mm diameter was used as a scaffold for keratinocytes in tissue culture inserts in a 24-well plate. These were cultured submerged for three days, and subsequently the medium level was lowered to allow air-exposure to induce terminal differentiation. After seven days of air exposure a fully stratified epidermis has developed that expresses all normal differentiation markers, but is negative for psoriasis-associated genes like CK16, SKALP/elafin and hBD-2. At this point a mixture of 10 ng/ml IL-1α, 5 ng/ml TNFα and 5 ng/ml IL-6 is added for 72 hours, which induces high expression levels of psoriasis-associated genes. The culture medium, 450 µl per culture, was harvested and changed at 24, 48 and 72 hours and used for hBD-2 ELISA. Two different donors for human keratinocytes were used.

### Statistics

All statistical analyses were performed using the Statistica software package version 7, Statsoft Inc. Analysis of variance was performed on log transformed data of serum hBD-2. Analysis of qPCR data was done on deltaCt values.

### Modelling of epidermal hBD-2 concentrations

Fick's law for mass transport was applied. For the diffusion coefficient (D) of hBD-2 we used 1.44×10^−10^ m^2^/sec as experimentally determined for bovine pancreatic trypsin inhibitor (BPTI) [37], a protein of similar mass and isoelectric point as hBD-2, or we used an approximation based on Graham's law using the known value for BSA (10^−10^ m^2^/sec). The hBD-2 production) in a reconstructed skin model stimulated with proinflammatory cytokines was used estimate the concentration in the epidermal compartment *in vitro*, as an approximation of the concentration in psoriatic epidermis. See supplemental text S1 and supplemental figure S1 for details.

## Results

### Expression of beta-defensin mRNA and protein in normal tissues and inflamed skin

**Figure 2:**
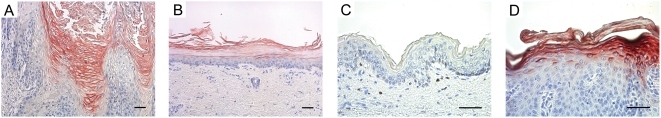
Immunolocalization of hBD-2 in human epithelia. Immunohistochemical staining of normal human tissues (tongue, plantar skin and trunk skin, A–C) with a polyclonal rabbit antiserum against recombinant hBD-2. Note that protein data largely follow the mRNA data demonstrating the absence in normal skin, low expression in tongue and plantar skin. Bar = 100 µm. Control sections stained with pre-immune serum were negative (not shown).

Although expression of beta-defensins in some human tissues has been documented before, we wanted to obtain a comprehensive picture of beta-defensin tissue distribution in order to estimate which specific tissues would contribute to systemic protein levels. We performed a qPCR analysis of two beta-defensin genes that are contained in the repeat on chromosome 8 (*DEFB4* and *DEFB103*) and one beta-defensin outside the repeat (*DEFB1*). Figure 1 shows that hBD-1 mRNA is widely expressed whereas significant levels of hBD-2 are predominantly found in oral epithelia. hBD-3 is expressed more broadly than hBD-2 as it is also found at low levels in normal epidermis and a few other tissues. As indicated in figure 1 (log scale) hBD-2 expression levels in lesional psoriatic epidermis are extremely high compared to any other tissue. hBD-2 mRNA expression in atopic dermatitis is also increased, compared to normal skin where it is undetectable. We analyzed the expression of hBD-2 at the protein level by immunohistochemistry. Figure 2A–E confirm the moderate expression levels in tongue and plantar epidermis, and the absence of expression in normal skin. The extremely high mRNA expression level in psoriatic epidermis was indeed reflected by high levels of protein as we have described before [10].

### Serum hBD-2 levels in healthy volunteers correlate with genomic copy number

**Figure 3:**
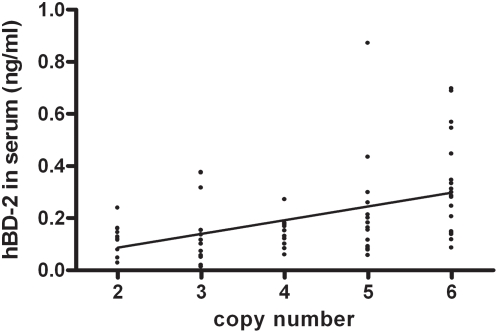
Correlation between serum hBD-2 protein levels and genomic copy number. Serum hBD-2 protein levels of 70 healthy controls (determined by ELISA) were plotted against the genomic copy number of the beta-defensin repeat on chromosome 8p23, as determined by MAPH, REDVR and PRT. A significant linear correlation was found. Pearson's R = 0.46 and p<7×10^−5^.

As a next step we wanted to make a quantitative determination of systemic defensin protein levels. Defensins are secreted proteins that will easily pass basal membranes, and we expected that epithelium-expressed defensins would reach the circulation and could be measured in serum by ELISA. On the basis of the limited expression of hBD-2 in normal human tissues, as demonstrated in figure 1, we expected low levels of protein in normal human serum. We developed and validated an ELISA that was sensitive and specific for hBD-2 to allow measurement of low serum hBD-2 levels. In addition, commercially available ELISA kits were used to measure hBD-2 and hBD-3 protein in serum. We have previously shown that most beta-defensin genes on 8p23.1 (including those that encode hBD-2 and hBD-3) are subject to copy number variation. So far no studies have been published that investigated the relation between copy number and protein expression. Here we selected 70 healthy individuals without inflammatory skin disease, that were typed for beta-defensin copy number using multiplex amplifiable probe hybridization (MAPH), restriction enzyme digest variant ratio (REDVR) and paralogue ratio test (PRT). In the normal population, copy number classes of 3 to 5 defensin repeats per diploid genome are the most prevalent. We selected individuals to include sufficient numbers of the more rare copy classes of 2 and 6 to allow analysis of correlation between genomic copy number and serum hBD-2 protein. Figure 3 shows that there is a modest but highly significant correlation between copy number and hBD-2 protein concentration in serum. No correlation between serum hBD-2 and age or gender was found. Low hBD-2 levels were found up to 0.80 ng/ml in these human sera (mean and SD: 0.21±0.17 ng/ml, see table 1), but clearly the variance is only partially explained by copy number. A similarly weak correlation (Pearson's R = 0.4, p = 0.001) between copy number and serum protein was found using a commercially available hBD-2 ELISA, although absolute levels of hBD-2 appeared to be higher for the commercial kit, which was probably due to the difference in standards used (not shown). hBD-3 serum levels of individuals without inflammatory skin disease were all below the detection limit (0.1 ng/ml). These data show that hBD-2 (but not hBD-3) can be measured in normal human serum, and that these uninduced levels are at least in part determined by genomic copy number.

### hBD-2 serum levels of psoriasis patients correlate with clinical severity

**Figure 4:**
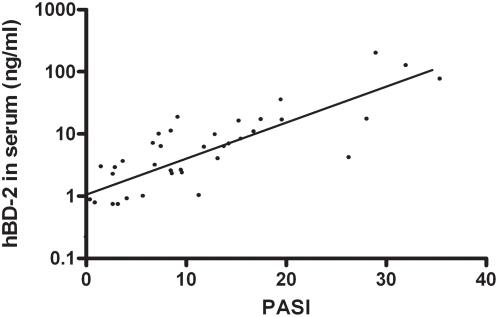
Correlation between serum hBD-2 protein and PASI score. Serum hBD-2 protein levels of 38 psoriasis patients of varying disease severity were plotted against their PASI score. A significant linear correlation was found. Pearson's R = 0.82, p<4×10^−10^.

Because of the modest expression of hBD-2 mRNA in most normal tissues we expected relatively low levels of hBD-2 protein in the circulation, as was indeed found by ELISA in control individuals (figure 3). In view of the huge increase of hBD-2 mRNA in lesional psoriatic epidermis, the inflamed skin of psoriasis patients was likely to cause increased systemic levels of hBD-2 protein. We measured hBD-2 in sera of 38 psoriasis patients in a range of clinical severity as determined by the Psoriasis Area and Severity Index (PASI) score (see table 1). We could indeed demonstrate high levels of serum hBD-2 in psoriasis patients, which were found to be about 400-fold increased in severely affected patients compared to healthy individuals (see table 1). A significant effect of disease severity on serum hBD-2 was found by analysis of variance (p<2×10^−6^). Figure 4 shows that there is a strong linear correlation between PASI score and the log-transformed serum hBD-2 concentration. Strongly increased levels were also found in the urine of psoriasis patients whereas hBD-2 could not be detected in urine of healthy controls. Urinary hBD-2 levels were showed strong interindividual variation (2.2+3.2 ng/ml). These findings suggest that increased systemic levels are derived from high cutaneous production. It is obvious from figure 4 that the clinical severity in psoriasis patients is a far stronger determinant of hBD-2 serum concentration than copy number for uninduced levels in healthy controls (figure 3). There were insufficient numbers of informative psoriasis patients from whom defensin copy numbers and PASI scores were available, to make a reliable estimate of the effect of copy number and disease severity on hBD-2 levels. Remarkably, only in a minority of psoriasis patients we could demonstrate hBD-3 levels that exceeded the detection limit of the assay (not shown).

To investigate if high systemic hBD-2 levels could be caused by inflammation per se, irrespective of the tissue localization, we compared serum hBD-2 levels in psoriasis patients with that of 40 patients with rheumatoid arthritis (RA). Low hBD-2 serum levels were found in RA patients with low to high disease activity and in RA patients that were in remission (see table 1). In the combined RA patients with moderate to high disease activity, a small but significant increase of serum hBD-2 was found compared to the control group (t-test, p<10^−5^). qPCR analysis of synovial tissue from RA patients with active disease showed that the synovium is unlikely to be the source of this small increase in hBD-2 protein, as no mRNA for hBD-2 could be detected (see figure 1). No significant correlation was found between serum hBD-2 and clinical severity as determined by Disease Activity Score (DAS28).

As previously reported [10], hBD-2 protein is expressed at low to undetectable levels (immunohistochemistry) in lesional atopic dermatitis, despite strong induction at the mRNA level. In sera from 8 patients with moderate to severe atopic dermatitis we found that, although serum hBD-2 levels were significantly higher than in control sera (t-test, p<10^−5^), they were far lower than in sera of psoriasis patients of similar disease severity (see table 1). A significant linear correlation between log-transformed serum hBD-2 and atopic dermatitis disease severity, as determined by SCORing Atopic Dermatitis (SCORAD), was found (Pearson r = 0.7, p<0.01), suggesting that also in atopic dermatitis increased systemic levels are derived from increased cutaneous production.

### Serum hBD-2 is a disease marker in individual psoriasis patients

**Figure 5:**
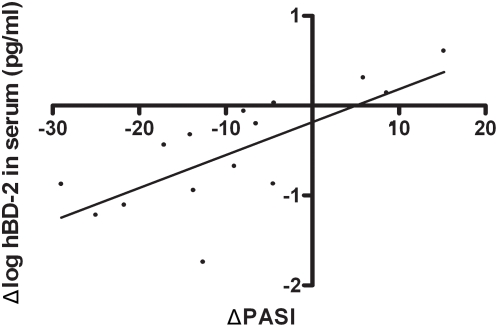
Correlation between the change in serum hBD-2 concentration and change in clinical score. Serum hBD-2 protein levels of 15 patients for which PASI scores and serum was available on two different occasions over a 6–18 week interval, were plotted against the change in PASI score (ΔPASI). A significant linear correlation was found. Pearson R = 0.74, p<0.002. Note that there was a decrease in serum hBD-2 in most patients that showed clinical improvement (negative ΔPASI) and an increase in serum hBD-2 in a few patients that showed exacerbation (positive ΔPASI).

As serum hBD-2 showed a correlation with disease activity over a group of psoriasis patients, we next investigated if hBD-2 levels correlate with clinical status of individual patients. Serum hBD-2 was measured in 15 patients that participated in clinical studies for which PASI scores and serum was available on two different occasions over a 6–18 week interval. Figure 5 shows that there is a strong correlation between the change in hBD-2 (ΔhBD-2) and change in PASI score (ΔPASI) in eleven patients that showed clinical improvement and four patients that showed exacerbation of disease.

### Estimation of *in vivo* hBD-2 concentration in psoriatic epidermis using *in vitro* reconstructed skin

**Figure 6:**
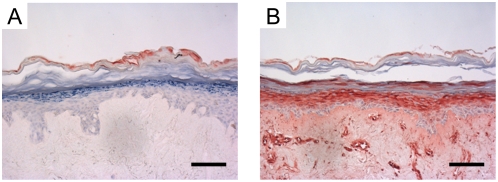
Induction of hBD-2 protein expression in reconstructed skin by proinflammatory cytokines. Expression of hBD-2 in 3-D reconstructed skin following stimulation with psoriasis-associated cytokines (10 ng/ml IL-1α, 5 ng/ml TNFα and 5 ng/ml IL-6) for 72 hours. Note that without stimulation there is no hBD-2 expression (A), whereas the cytokine mixture induces high expression levels that are secreted into the underlying culture medium (147 ng/ml in 24 h). Part of the hBD-2 protein remains adsorbed to the dermal matrix as witnessed by the staining of structures in the dermis (B). Bar = 100 µm.

The high serum levels of hBD-2 in psoriasis patients (up to 190 ng/ml), which we interpret to be derived from local production by the keratinocytes, suggest that the concentrations to which keratinocytes and infiltrated cells in the epidermis and papillary dermis are exposed must be several orders of magnitude higher. Based on *in vitro* studies, the concentrations required to have a relevant biological effect would vary between 0.1 and 100 µg/ml. Because these local tissue concentrations are difficult to estimate directly from *in vivo* data, we measured the production of hBD-2 in reconstructed skin as a model for human epidermis. Human reconstructed skin equivalents were obtained as described previously [38], and stimulated with IL-1α, TNF-α and IL-6. This cytokine mixture was recently found to be optimal for induction of a psoriatic phenotype *in vitro*, as witnessed by high expression levels of CK16, SKALP/elafin and hBD-2 [36]. qPCR analysis of isolated epidermal sheets indicated that hBD-2 mRNA levels (determined as deltaCt values) in these cultures were much higher than in keratinocytes from submerged cultures (not shown), and reached similar high values as found in epidermal sheets from lesional psoriatic skin (see figure 1). Figure 6 shows hBD-2 protein expression by differentiated keratinocytes in the reconstructed skin model stimulated with pro-inflammatory cytokines. We collected culture medium from cytokine-stimulated reconstructed skin of two donors during 24 hours starting two days after addition of the cytokine mix. Using ELISA we found that these cultures, consisting of 8 mm diameter skin constructs, secreted approximately 66±19 ng hBD-2 per 24 hours into the tissue culture medium (mean and SD of four cultures). We derived the local epidermal concentration using an approximation based on the known diffusion coefficient of a small cationic protein in aqueous solution (see supplemental materials for assumptions and dimensions of the system, used for modelling). Alternatively, a theoretical value for the diffusion coefficient was obtained based on Graham's law (see supplemental materials). The most conservative estimation of the hBD-2 local concentration in the compartment of the epidermis was 1.2 mg/ml (0.3 mM), which is far higher than concentrations required for *in vitro* antimicrobial activity, or those reported for chemotactic activity towards T-cells and dendritic cells.

## Discussion

Psoriasis is associated with increased beta-defensin genomic copy number but no quantitative information on defensin protein expression *in vivo*, as a function of copy number or disease status was available. We now present evidence that both genomic copy number and epidermal activation by psoriasis-associated cytokines determine expression levels of hBD-2. We conclude that these increased circulating hBD-2 levels in patients are derived from cutaneous production, indicating that local hBD-2 concentrations in psoriatic epidermis are likely to be extremely high. Extrapolation from an *in vitro* model system suggests that the concentration in the epidermis or papillary dermis (0.3 mM) by far exceeds the concentrations required to be antimicrobial (2.5 to 25 µM) and chemotactic *in vitro* (250 nM) as described previously by others [9], [39].

A number of studies have reported expression of human beta-defensins in various tissues, including normal skin, inflamed skin and non-cutaneous epithelia, using semi-quantitative RT-PCR or immunohistochemistry. Here we have quantified circulating levels of hBD-2 and hBD-3 by ELISA in healthy volunteers and patients with inflammatory diseases. To assess which of the normal human epithelia would contribute to steady state systemic protein levels, we first performed a comprehensive qPCR analysis of the three major epidermal defensins, hBD-1, -2 and -3 in a large panel of human tissues. As shown in figure 1, hBD-1 mRNA is widely expressed, and is not subject to regulation by inflammation, as it is not highly induced in psoriatic skin. Measurement of hBD-1 protein in serum would have provided a reference value for a beta-defensin that is expected to be rather constant as it is not present on the psoriasis-associated repeat. We were however unsuccessful in measuring hBD-1 protein by ELISA (not shown). hBD-2 mRNA appears to be expressed constitutively by a few tissues (e.g. oral epithelia), and was indeed found at the protein level albeit weakly (figure 2). hBD-2 is absent from normal trunk skin, but it is expressed at low levels in plantar skin. Consequently, serum hBD-2 levels in healthy individuals are very low (less than 1 ng per ml). In addition to our own ELISA (recombinant hBD-2 for calibration), we also used a commercial hBD-2 ELISA kit that uses synthetic hBD-2 for calibration. Although there was a good correlation between the data of both assays (Pearson r = 0.9), the commercial ELISA yielded apparent hBD-2 serum concentrations 10-fold higher than our ELISA. We found that this was caused by a difference in immunoreactivity of the calibration standards used. We used the values obtained with recombinant hBD-2 throughout this paper, as a conservative estimate of hBD-2 concentration.

As shown in figure 3, genomic copy number only explains part of the variance in serum hBD-2 levels. This is unlikely to be caused by experimental error. We considered sampling error as a potential cause, but we did not find variation in serum hBD-2 level depending on the time of blood sample collection (early morning versus late afternoon). Longitudinal data showed only minor fluctuation of serum hBD-2 levels in blood taken with two-year intervals (not shown). We speculate that the large variation of protein levels in each copy class could be caused by variation in expression regulation, or alternatively, by genetic variation in the defensin copies themselves. Although there is currently no information on the existence of non-functional gene copies within the repeat, this would obviously affect the gene-protein correlation, and it could also affect the observed association with psoriasis, which clearly requires further investigation.

Although our findings indicate that uninduced hBD-2 production is subject to gene dosage effects, the high systemic serum levels in psoriasis patients are caused by disease activity itself. We think this indicates that hBD-2 can be used as an objective, quantitative marker for disease activity in psoriasis. There are very few reliable serum markers for disease activity in psoriasis. Candidate proteins that are overexpressed in lesional epidermis include SKALP/elafin and psoriasin/S100A7, but only SKALP/elafin was found to correlate with disease activity [40], [41]. Previous studies have suggested that conventional surrogate markers for disease activity in rheumatoid arthritis and psoriatic arthritis, such as erythrocyte sedimentation rate and levels of C-reactive protein (CRP) are not useful for chronic plaque psoriasis [42]. For this reason we looked at the correlation between PASI scores and CRP in a cohort of our own patients that were monitored over time. We did not find a significant correlation between CRP and PASI for 1305 patient visits (data not shown), again suggesting that serum hBD-2 could be a convenient way to monitor disease activity or therapeutic effects.

At the tissue level, hBD-2 secretion will be determined both by copy number and by stimulation with proinflammatory cytokines. Our data also suggest that disease activity in atopic dermatitis is correlated with serum hBD-2. However, the epidermal expression in atopic dermatitis is far lower than in psoriasis [10], [11], presumably resulting from the distinct cytokine environments of these diseases [8], [28]. We could indeed show that Th2 cytokines do not induce hBD-2 expression in cultured keratinocytes [18] and that Th2 cytokines repress the Th1 induced expression (not shown here), as was previously shown for hBD-3 [43]. An additional level of regulation of hBD-2 expression is the responsiveness of cells to pro-inflammatory cytokines. We have recently shown that cell-autonomous differences exist between keratinocytes of psoriasis patients, atopic dermatitis patients and controls with respect to innate immune responses following Th1 cytokine stimulation [18] . We would speculate that there are apparently three mechanisms that contribute to high levels of hBD-2 in psoriatic epidermis: a dominant Th1 or Th17 cytokine profile, increased cell-autonomous responsiveness to cytokine stimulation, and high defensin genomic copy number. Although defensins are endowed with cytokine-like properties themselves, there is no information on a possible autocrine effect of hBD-2 on keratinocytes whereby it would induce or sustain its own production. This is clearly an area worth investigating.

Several studies have demonstrated the biological activities of human and mouse beta-defensins, such as antimicrobial activity [9], chemotaxis of T-cells and dendritic cells [44], and activation of TLR4 [45]. In general, many antimicrobial proteins appear to be endowed with other immune-modulatory activities [46]–[48]. These findings have raised the intriguing possibility that beta-defensins are part of the innate immune system that provides a link between the epithelium and the adaptive immune system. This would fit the emerging concept of inflammatory epithelial diseases [14] that is supported by findings on the genetics of atopic dermatitis [15], Crohn's disease [49] and celiac disease [50]. This would also explain the clinical observation that both T-cell directed drugs (cyclosporine A, UVB, some biologicals) and keratinocyte-directed drugs (retinoids, vitamin D3 derivatives) are effective in the treatment of psoriasis.

The defensin concentrations used in *in vitro* studies to demonstrate their biological activity and their relevance for the *in vivo* situation, should be interpreted with some caution. For example, the antimicrobial activity of hBD-2 can only be demonstrated at low salt conditions [51], at least in low micromolar concentrations. Notwithstanding these considerations, we attempted to make an estimate of the actual hBD-2 concentration in an active psoriatic lesion. Although there are some caveats associated with modelling of the *in vivo* tissue concentrations based on an *in vitro* reconstructed skin model, it is clear that the hBD-2 levels in epidermis and papillary dermis are well above the minimal concentrations that would cause a biological effect in an experimental setting, even allowing for an error of one order of magnitude. Remarkably, we found very little hBD-3 protein in the circulation. Only in a few psoriasis patients we observed detectable levels up to 0.5 ng/ml, which is 160-fold lower than the hBD-2 concentration in patients with severe psoriasis. Whether this also reflects low hBD-3 protein production in the epidermis requires further investigation.

Our previous finding on the genetic association of psoriasis with the defensin cluster did not allow discrimination between the seven defensins or defensin-like genes contained in the repeated segment. On the basis of available expression data we predicted that hBD-2 and hBD-3 would be the best candidates. Here we provide further evidence that, based on copy number dependent and disease-specific gene expression, high cutaneous protein levels and its known proinflammatory properties, hBD-2 is one of the strongest psoriasis candidate genes contained in the beta defensin cluster on chromosome 8.

## Supporting Information

Table S1 Primers for qPCR
(0.03 MB DOC)

Figure S1 Diffusion model
(0.35 MB DOC)

Text S1 Calculation of hBD-2 mass transport in reconstructed skin model
(0.03 MB DOC)

## References

[1] Bowcock AM, Krueger JG (2005). Getting under the skin: the immunogenetics of psoriasis.. Nat Rev Immunol.

[2] Nickoloff BJ, Qin JZ, Nestle FO (2007). Immunopathogenesis of psoriasis.. Clin Rev Allergy Immunol.

[3] Nair RP, Stuart PE, Nistor I, Hiremagalore R, Chia NV (2006). Sequence and haplotype analysis supports HLA-C as the psoriasis susceptibility 1 gene.. Am J Hum Genet.

[4] Cargill M, Schrodi SJ, Chang M, Garcia VE, Brandon R (2007). A large-scale genetic association study confirms IL12B and leads to the identification of IL23R as psoriasis-risk genes.. Am J Hum Genet.

[5] Capon F, Di Meglio P, Szaub J, Prescott NJ, Dunster C (2007). Sequence variants in the genes for the interleukin-23 receptor (IL23R) and its ligand (IL12B) confer protection against psoriasis.. Hum Genet.

[6] Menssen A, Trommler P, Vollmer S, Schendel D, Albert E (1995). Evidence for an antigen-specific cellular immune response in skin lesions of patients with psoriasis vulgaris.. J Immunol.

[7] Nestle FO, Conrad C, Tun-Kyi A, Homey B, Gombert M (2005). Plasmacytoid predendritic cells initiate psoriasis through interferon-{alpha} production.. J Exp Med.

[8] Nickoloff BJ, Xin H, Nestle FO, Qin JZ (2007). The cytokine and chemokine network in psoriasis.. Clin Dermatol.

[9] Harder J, Bartels J, Christophers E, Schroder JM (1997). A peptide antibiotic from human skin.. Nature.

[10] de Jongh GJ, Zeeuwen PL, Kucharekova M, Pfundt R (2005). High expression levels of keratinocyte antimicrobial proteins in psoriasis compared with atopic dermatitis.. J Invest Dermatol.

[11] Ong PY, Ohtake T, Brandt C, Strickland I, Boguniewicz M (2002). Endogenous antimicrobial peptides and skin infections in atopic dermatitis.. N Engl J Med.

[12] Nomura I, Gao B, Boguniewicz M, Darst MA, Travers JB (2003). Distinct patterns of gene expression in the skin lesions of atopic dermatitis and psoriasis: a gene microarray analysis.. J Allergy Clin Immunol.

[13] Nomura I, Goleva E, Howell MD, Hamid QA, Ong PY (2003). Cytokine milieu of atopic dermatitis, as compared to psoriasis, skin prevents induction of innate immune response genes.. J Immunol.

[14] Cookson W (2004). The immunogenetics of asthma and eczema: a new focus on the epithelium.. Nat Rev Immunol.

[15] Palmer CN, Irvine AD, Terron-Kwiatkowski A, Zhao Y, Liao H (2006). Common loss-of-function variants of the epidermal barrier protein filaggrin are a major predisposing factor for atopic dermatitis.. Nat Genet.

[16] Hollox EJ, Huffmeier U, Zeeuwen PL, Palla R, Lascorz J (2008). Psoriasis is associated with increased beta-defensin genomic copy number.. Nat Genet.

[17] de Cid R, Riveira-Munoz E, Zeeuwen PL, Robarge J, Liao W (2009). Deletion of the late cornified envelope LCE3B and LCE3C genes as a susceptibility factor for psoriasis.. Nat Genet.

[18] Zeeuwen PL, de Jongh GJ, Rodijk-Olthuis D, Kamsteeg M, Verhoosel RM (2008). Genetically programmed differences in epidermal host defense between psoriasis and atopic dermatitis patients.. PLoS ONE.

[19] Ganz T (2003). Defensins: antimicrobial peptides of innate immunity.. Nat Rev Immunol.

[20] Yang D, Chertov O, Bykovskaia SN, Chen Q, Buffo MJ (1999). Beta-defensins: linking innate and adaptive immunity through dendritic and T cell CCR6.. Science.

[21] Niyonsaba F, Ogawa H, Nagaoka I (2004). Human beta-defensin-2 functions as a chemotactic agent for tumour necrosis factor-alpha-treated human neutrophils.. Immunology.

[22] Niyonsaba F, Iwabuchi K, Matsuda H, Ogawa H, Nagaoka I (2002). Epithelial cell-derived human beta-defensin-2 acts as a chemotaxin for mast cells through a pertussis toxin-sensitive and phospholipase C-dependent pathway.. Int Immunol.

[23] Hollox EJ, Armour JA, Barber JC (2003). Extensive normal copy number variation of a beta-defensin antimicrobial-gene cluster.. Am J Hum Genet.

[24] Harder J, Schroder JM (2005). Antimicrobial peptides in human skin.. Chem Immunol Allergy.

[25] Liu AY, Destoumieux D, Wong AV, Park CH, Valore EV (2002). Human beta-defensin-2 production in keratinocytes is regulated by interleukin-1, bacteria, and the state of differentiation.. J Invest Dermatol.

[26] Henseler T, Christophers E (1995). Disease concomitance in psoriasis.. J Am Acad Dermatol.

[27] Gallo RL, Murakami M, Ohtake T, Zaiou M (2002). Biology and clinical relevance of naturally occurring antimicrobial peptides.. J Allergy Clin Immunol.

[28] Leung DY, Bieber T (2003). Atopic dermatitis.. Lancet.

[29] Hoogendoorn EH, Hermus AR, de Veght F, Ross HA, Verbeek AL (2006). Thyroid function and prevalence of anti-thyroperoxidase antibodies in a population with borderline sufficient iodine intake: influences of age and sex.. Clin Chem.

[30] Schmitt J, Wozel G (2005). The psoriasis area and severity index is the adequate criterion to define severity in chronic plaque-type psoriasis.. Dermatology.

[31] Kunz B, Oranje AP, Labreze L, Stalder JF, Ring J (1997). Clinical validation and guidelines for the SCORAD index: consensus report of the European Task Force on Atopic Dermatitis.. Dermatology.

[32] van Riel PL, Schumacher HR (2001). How does one assess early rheumatoid arthritis in daily clinical practice?. Best Pract Res Clin Rheumatol.

[33] Alkemade HA, Molhuizen HO, van Vlijmen-Willems IM, van Haelst UJ, Schalkwijk J (1993). Differential expression of SKALP/Elafin in human epidermal tumors.. Am J Pathol.

[34] Livak KJ, Schmittgen TD (2001). Analysis of relative gene expression data using real-time quantitative PCR and the 2(-Delta Delta C(T)) Method.. Methods.

[35] Armour JA, Palla R, Zeeuwen PL, den Heijer M, Schalkwijk J (2007). Accurate, high-throughput typing of copy number variation using paralogue ratios from dispersed repeats.. Nucleic Acids Res.

[36] Tjabringa G, Bergers M, van Rens D, de Boer R, Lamme E (2008). Development and validation of human psoriatic skin equivalents.. Am J Pathol.

[37] Gallagher WH, Woodward CK (1989). The concentration dependence of the diffusion coefficient for bovine pancreatic trypsin inhibitor: a dynamic light scattering study of a small protein.. Biopolymers.

[38] Schalkwijk J, Lamme E, de Jongh G, Zeeuwen P, Bergers M (2006). Psoriatic Skin Equivalents..

[39] Yang D, Chen Q, Chertov O, Oppenheim JJ (2000). Human neutrophil defensins selectively chemoattract naive T and immature dendritic cells.. J Leukoc Biol.

[40] Alkemade HA, de Jongh GJ, Arnold WP, van de Kerkhof PC, Schalkwijk J (1995). Levels of skin-derived antileukoproteinase (SKALP)/elafin in serum correlate with disease activity during treatment of severe psoriasis with cyclosporin A.. J Invest Dermatol.

[41] Anderson KS, Wong J, Polyak K, Aronzon D, Enerback C (2008). Detection of psoriasin/S100A7 in the sera of patients with psoriasis..

[42] Laurent MR, Panayi GS, Shepherd P (1981). Circulating immune complexes, serum immunoglobulins, and acute phase proteins in psoriasis and psoriatic arthritis.. Ann Rheum Dis.

[43] Howell MD, Boguniewicz M, Pastore S, Novak N, Bieber T (2006). Mechanism of HBD-3 deficiency in atopic dermatitis.. Clin Immunol.

[44] Yang D, Biragyn A, Kwak LW, Oppenheim JJ (2002). Mammalian defensins in immunity: more than just microbicidal.. Trends Immunol.

[45] Biragyn A, Ruffini PA, Leifer CA, Klyushnenkova E, Shakhov A (2002). Toll-like receptor 4-dependent activation of dendritic cells by beta-defensin 2.. Science.

[46] Di Nardo A, Braff MH, Taylor KR, Na C, Granstein RD (2007). Cathelicidin antimicrobial peptides block dendritic cell TLR4 activation and allergic contact sensitization.. J Immunol.

[47] Lande R, Gregorio J, Facchinetti V, Chatterjee B, Wang YH (2007). Plasmacytoid dendritic cells sense self-DNA coupled with antimicrobial peptide.. Nature.

[48] Braff MH, Hawkins MA, Di Nardo A, Lopez-Garcia B, Howell MD (2005). Structure-function relationships among human cathelicidin peptides: dissociation of antimicrobial properties from host immunostimulatory activities.. J Immunol.

[49] Fellermann K, Stange DE, Schaeffeler E, Schmalzl H, Wehkamp J (2006). A chromosome 8 gene-cluster polymorphism with low human Beta-defensin 2 gene copy number predisposes to crohn disease of the colon.. Am J Hum Genet.

[50] Monsuur AJ, de Bakker PI, Alizadeh BZ, Zhernakova A, Bevova MR (2005). Myosin IXB variant increases the risk of celiac disease and points toward a primary intestinal barrier defect.. Nat Genet.

[51] Bals R, Wang X, Wu Z, Freeman T, Bafna V (1998). Human beta-defensin 2 is a salt-sensitive peptide antibiotic expressed in human lung.. J Clin Invest.

