# Supplementary material for: β-Defensin-2 Protein Is a Serum Biomarker for Disease Activity in Psoriasis and Reaches Biologically Relevant Concentrations in Lesional Skin
Source: PLoS One. 2009 Mar 6;4(3):e4725. doi: 10.1371/journal.pone.0004725 (PMC2649503; doi:10.1371/journal.pone.0004725)
Supplement: Table S1 — Primers for qPCR (0.03 MB DOC) [file pone.0004725.s001.doc]

**Table S1: primers for qPCR**

|  | Forward primer (5' > 3') | Reverse primer (5' > 3') | E^*^ |
| --- | --- | --- | --- |
| hBD-1 | atggcctcaggtggtaactttc | cacttggccttccctctgtaac | 2.00 |
| hBD-2 | gatgcctcttccaggtgttttt | ggatgacatatggctccactctt | 1.97 |
| hBD-3 | gtgaagcctagcagctatgaggat | tgattcctccatgacctggaa | 2.06 |
| RPLP0 | caccattgaaatcctgagtgatgt | tgaccagcccaaaggagaag | 2.00 |

**^*^E is efficiency as fold increase in fluorescence per PCR cycle**
