# Supplementary figures and images for: β-Defensin-2 Protein Is a Serum Biomarker for Disease Activity in Psoriasis and Reaches Biologically Relevant Concentrations in Lesional Skin
Source: PLoS One. 2009 Mar 6;4(3):e4725. doi: 10.1371/journal.pone.0004725 (PMC2649503; doi:10.1371/journal.pone.0004725)

**Figure S1: diffusion model**


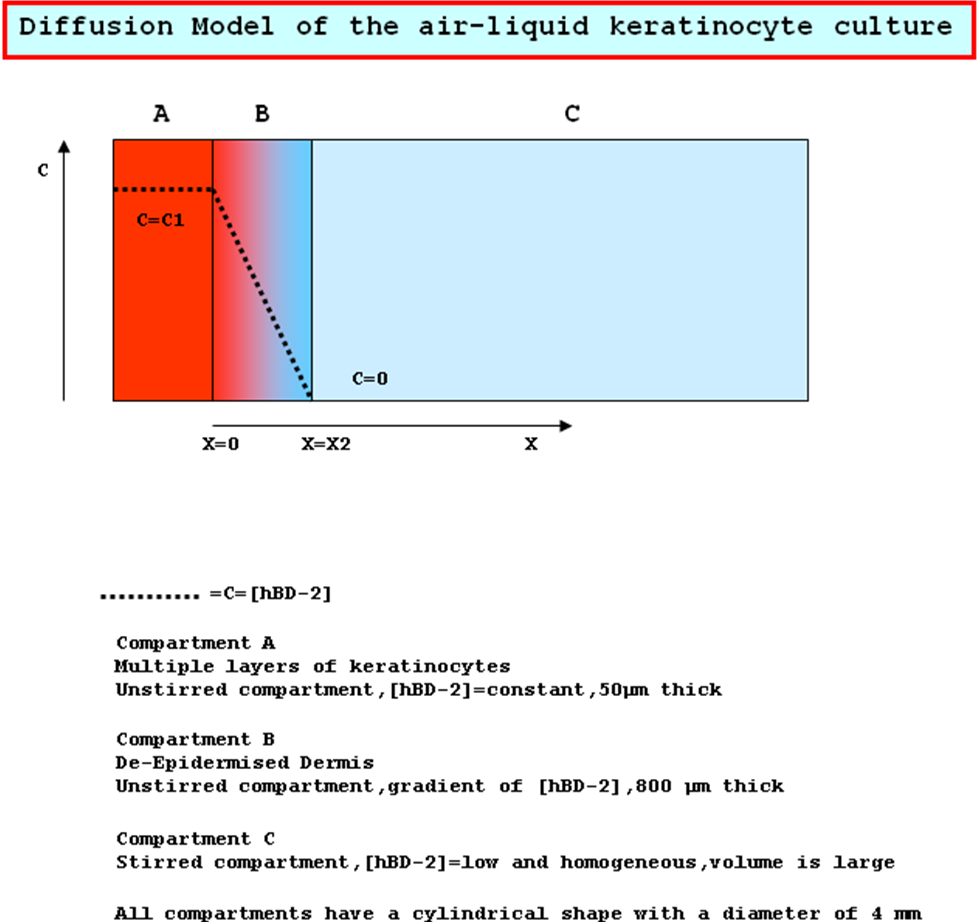


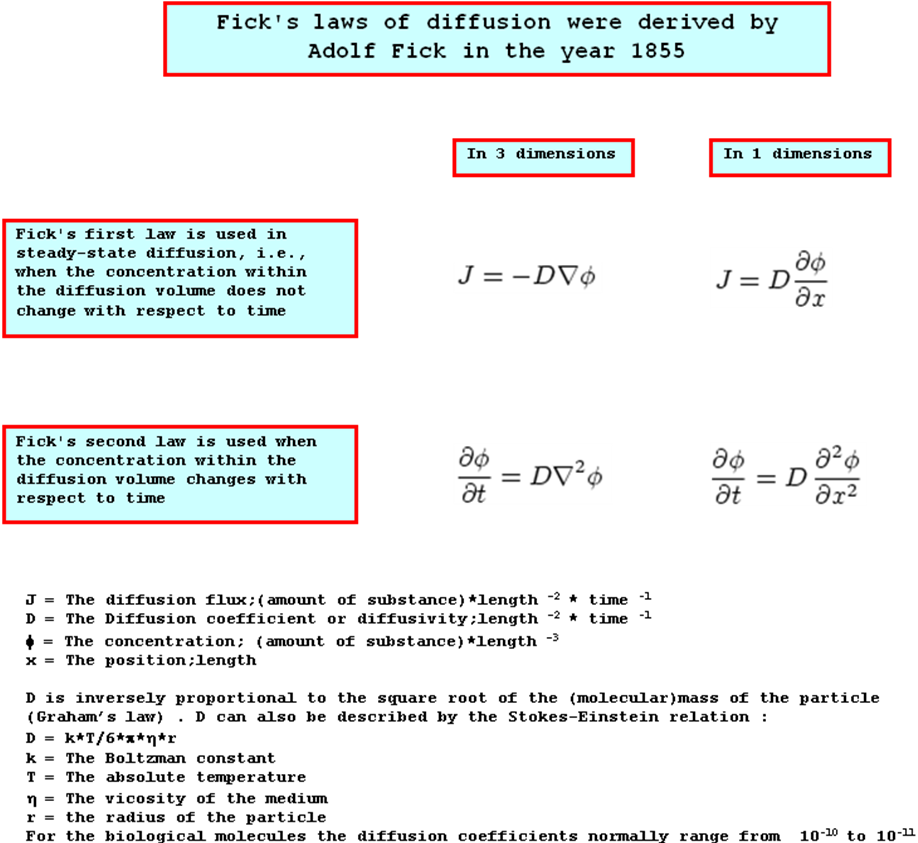

Supplement: Figure S1 — Diffusion model (0.35 MB DOC) [file pone.0004725.s002.doc]
