# Supplementary material for: β-Defensin-2 Protein Is a Serum Biomarker for Disease Activity in Psoriasis and Reaches Biologically Relevant Concentrations in Lesional Skin
Source: PLoS One. 2009 Mar 6;4(3):e4725. doi: 10.1371/journal.pone.0004725 (PMC2649503; doi:10.1371/journal.pone.0004725)
Supplement: Text S1 — Calculation of hBD-2 mass transport in reconstructed skin model (0.03 MB DOC) [file pone.0004725.s003.doc]

**Text S1: calculation of hBD-2 mass transport in reconstructed skin model**

Supplemental text 1

We apply Fick’s First law, because [hBD-2] is not a function of time. We use the condition only in 1 dimension, because [hBD-2] changes only in the X-direction. So:

Mass transport hBD-2 = J=dm/dt=-D*dc/dx=a_0_=constant --- 1)

or dc/dx=-a_0_/D

or dc=-a_0_/D*dx

or c=∫-a_0_/D*dx

or c=-a_0_/D*x+a_1_ ---2)

First boundary condition for x=0 c=c_1_ ---3)

Second boundary condition for x=x_2_ c=0 ---4)

2) + 3) => c_1_=-a_0_/D*0+a_1_ => a_1_=c_1_ ---5)

2) + 4) + 5) => 0=-a_0_/D*x_2_+c_1_ => a_0_=c_1_*D/x_2_ ---6)

2) + 5) + 6) => c=-c_1_/x_2_*x+c_1_ ---7)

The graph of this function is plotted in compartment B

1. + 7) => J=dm/dt=-D*dc/dx=-D*d(-c_1_/x_2_*x+c_1_)/dx

or J=D/x_2_*c_1_ per surface unit

When we ignore the boundary conditions and the DED has a surface O, than:

Mass transport of hBD-2 = D*O/x_2_ * c_1_ in Mol/sec

D = the Diffusion constant of the DED

O = the Surface of the DED

x_2_ = the thickness of the DED

c_1_ = the [hBD-2] we wanted to know
